# Treating Patients With Locally Advanced Squamous Cell Carcinoma of the Head and Neck Unsuitable to Receive Cisplatin-Based Therapy

**DOI:** 10.3389/fonc.2019.01522

**Published:** 2020-01-22

**Authors:** Sandro V. Porceddu, Florian Scotté, Matti Aapro, Satu Salmio, Ana Castro, Vincent Launay-Vacher, Lisa Licitra

**Affiliations:** ^1^University of Queensland, Princess Alexandra Hospital, Woolloongabba, QLD, Australia; ^2^Department of Medical Oncology and Supportive Care, Hôpital Foch, Suresnes, France; ^3^Genolier Cancer Center, Genolier, Switzerland; ^4^Merck KGaA, Darmstadt, Germany; ^5^Lenitudes Medical Center & Research, Santa Maria da Feira, Portugal; ^6^Service ICAR–Department of Nephrology, Hôpital Pitié-Salpêtrière, Paris, France; ^7^Fondazione IRCCS Istituto Nazionale Tumori and University of Milan, Milan, Italy

**Keywords:** cisplatin, locally advanced squamous cell carcinoma of the head and neck, toxicity, cetuximab, radiotherapy, chemotherapy

## Abstract

Concurrent chemoradiotherapy with high-dose cisplatin (100 mg/m^2^ every 3 weeks) is the preferred regimen with curative intent for patients with unresected locally advanced squamous cell carcinoma of the head and neck (LA SCCHN). This treatment is associated with acute and late toxicities, including myelosuppression, severe nausea/vomiting, irreversible renal failure, hearing loss, and neurotoxicity. Because of cisplatin's safety profile, treatment adherence to high-dose cisplatin can be suboptimal. Patients commonly receive less than the total cumulative target dose of 300 mg/m^2^ or the minimum recommended dose of 200 mg/m^2^, which can have a negative impact on locoregional control and survival. Alternatively, cetuximab plus radiotherapy may be most suitable for patients at high risk of non-adherence to high-dose cisplatin. We discuss the baseline characteristics dictating the unsuitability/borderline unsuitability of cisplatin and the available alternative evidence-based treatment regimens for patients with LA SCCHN. We non-systematically reviewed published phase II and III trials and retrospective analyses of high-dose cisplatin-based chemoradiation in LA SCCHN conducted between 1987 and 2018, focusing on recent key phase III studies. We defined the baseline characteristics and associated prescreening tests to determine unsuitability and borderline unsuitability for high-dose cisplatin in combination with radiotherapy in patients with LA SCCHN. Patients with any pre-existing comorbidities that may be exacerbated by high-dose cisplatin treatment can be redirected to a non-cisplatin-based option to minimize the risk of treatment non-adherence. High-dose cisplatin plus radiotherapy remains the preferred treatment for fit patients with unresected LA SCCHN; patients who are unsuitable or borderline unsuitable for high-dose cisplatin could be identified using available tests for potential comorbidities and should be offered alternative treatments, such as cetuximab plus radiotherapy.

## Introduction

Squamous cell carcinoma of the head and neck (SCCHN) is among the most frequent cancers worldwide (1). Although recurrent and/or metastatic disease has a poor prognosis, earlier-stage, and non-metastatic locally advanced (LA) SCCHN remains potentially curable. Prognosis strongly depends on factors such as the primary site, disease stage, and human papillomavirus (HPV) infection status in oropharyngeal cancer (OPC), with the intricacies of personalized treatment still being evaluated.

Radiotherapy plus concomitant chemotherapy for LA SCCHN (stage III-IV) has been shown to improve 5 year absolute survival by 6.5% compared with locoregional treatment alone, as reported in a meta-analysis (2). However, approximately half of the patients receiving chemoradiotherapy develop recurrence (3), highlighting the unmet medical need for this patient population.

According to the guidelines of the European Society for Medical Oncology (ESMO; currently being updated) and the National Comprehensive Cancer Network, concurrent cisplatin-based chemoradiotherapy is the preferred treatment for fit patients with unresectable LA SCCHN and for those with resectable LA SCCHN but with a poor prognosis, functional loss, or high-risk features in the post-operative setting (i.e., extranodal extension and/or positive margins) (4, 5). The recommended administration schedule for cisplatin in the LA setting is three cycles of 100 mg/m^2^ every 3 weeks (high-dose cisplatin) with conventional fractionation radiotherapy or two cycles with altered fractionation radiotherapy (4, 6).

The treatment efficacy of cisplatin-based chemoradiotherapy appears to correlate with the cumulative cisplatin dose received by the patient. Specifically, a multivariable analysis showed that overall survival (OS) is significantly lower in patients with HPV-negative LA SCCHN (including OPC, laryngo-hypopharyngeal cancer, and carcinoma of unknown primary) who receive a cumulative cisplatin dose of ≤200 mg/m^2^ than in those who receive >200 mg/m^2^ (7). However, cisplatin is associated with both acute and late, often irreversible toxicities, which manifest as detrimental short- and long-term complications for patients. Thus, treatment adherence to high-dose cisplatin is low, and administration of the necessary cisplatin dose to effectively treat LA SCCHN is not feasible for many patients (8). Indeed, large-scale trials from the past two decades using conventional fractionation showed that only 61–85% of patients with LA SCCHN were able to receive three 100 mg/m^2^-doses of cisplatin (6, 9, 10). Furthermore, a retrospective European study assessing compliance with cisplatin plus radiotherapy in the real-world setting (COMPLY) demonstrated that a cumulative dose of >200 mg/m^2^ was reached in only 45% of patients receiving the high-dose regimen (8), and a retrospective study by Espeli et al. (11) showed that ≈50% of patients are able to complete the intended treatment of three cycles of high-dose cisplatin.

Unlike the population commonly enrolled into clinical trials, patients with LA SCCHN in the real-world clinical setting often present with more challenging baseline characteristics and risk factors [e.g., poor Eastern Cooperative Oncology Group performance status (ECOG PS) and comorbidities]. Unfortunately, no standardization of supportive care during chemoradiotherapy exists, and patient volume at treatment centers can affect the quality of treatment selection and supportive care (12). Therefore, it is important to compile a set of risk factors that pose a challenge to compliance with high-dose cisplatin and a comprehensive picture of alternative options to consider during treatment decision making.

In this review, we discuss risk factors associated with high-dose cisplatin that can enable physicians to identify which patients with LA SCCHN are eligible, ineligible, or eligible with a high risk of treatment non-compliance—the so-called “borderline unsuitable”—for concurrent high-dose cisplatin chemoradiotherapy with curative intent. Most of the data described here emerged before the distinct clinical entity of HPV-associated OPC was fully understood and when the proportion of patients with HPV-associated OPC enrolled in clinical trials was likely lower than that in contemporary cohorts. Therefore, the recent results of phase III studies of HPV-positive OPC exploring the clinical outcomes of cisplatin plus radiotherapy vs. alternative regimens are important.

## Cisplatin-Associated Toxicities That Can Compromise Treatment Compliance

The addition of high-dose cisplatin to radiotherapy both exacerbates known radiotherapy-associated adverse events (AEs) and causes specific cisplatin-associated AEs (Table 1), which are dose dependent. Nausea, vomiting, ototoxicity, nephrotoxicity, and neurotoxicity are also observed with cisplatin treatment (monotherapy or in combination) (36). Furthermore, these toxicities are cumulative, dose dependent, are often non-reversible (except nausea and vomiting), and can involve extensive injury to poorly regenerating or non-regenerating organs, resulting in exacerbation of pre-existing conditions and potentially a permanent impact on the quality of life (QOL) of cured patients (Table 2) (10, 11, 13, 14, 37).

**Table 1:** Cisplatin chemoradiotherapy–associated acute toxicities in head and neck cancer (9–11, 13–29).

**Induced toxicity^a^**	**Three weekly (100 mg/m**^****2****^**), %**		**Weekly (30–50 mg/m**^****2****^**), %**	
	**Any grade**	**Grade ≥3**	**Any grade**	**Grade ≥3**
**Reversible**				
Hematologic^b^	46–100	4–78	50–100	6–38
Mucositis/stomatitis	83–100	4–62	87–100	28–75
Dermatitis	90–100	0–56	89–100	1–35
Pharynx/esophagus/respiratory tract	100	6–54	91–100	22–54
Nausea/vomiting^b^	22–100	4–40	8–100	0–21
Gastrointestinal (not specified)^b^	33–100	3–32	30–38	3–20
Dysphagia	62–100	6–30	60–93	4–7
12-month FT dependency	19–20	NR	16	NR
Infection	24	0–20	NR	NR
Xerostomia/salivary gland^c^	79–100	0–15	79–88	0
Taste alteration	62–100	11	NR	NR
Constipation	4–32	0–4	4	4
Diarrhea^d^	4–7	1–2	NR	NR
Irreversible				
Neurotoxicity^b^^,^^d^	4–8	0–10	4–13	0
Nephrotoxicity^b^^,^^d^	7–75	0–8	3–35	0–3
Ototoxicity^b^^,^^d^	4–34	0–2	29	0

a *Reported in at least two of the analyzed publications*.

b *Also reported for treatment with cisplatin administered alone (30–33)*.

c *Can be irreversible in some cases (34)*.

d *Can be reversible on rare occasions (35)*.

*FT, feeding tube; NR, not reported*.

**Table 2:** Selected cisplatin chemoradiotherapy–associated late toxicities in head and neck cancer (10, 11, 13, 14, 35, 37–44).

**Cisplatin-induced toxicity**	**Grade ≥3, %**	**Grade ≥1, %**	**Cumulative dose,^**a**^ mg/m^**2**^**	**Risk factors**	**Recommended pre-enrollment tests**
Neurotoxicity	0–3	10	≥300	History of drug and alcohol use, diabetes, high serum creatinine levels, age	Vibration perception test (128 Hz tuning fork on upper and lower limbs), deep tendon reflex test
Nephrotoxicity	0–2	30–67	>50	Low ECOG PS, regular use of non-steroidal anti-inflammatory drugs, age, smoking, hypoalbuminemia, preexisting abnormal renal function (GFR <60 mL/min)	CCR, serum creatinine,^b^ or GFR
Ototoxicity	0–3	10	>60	Preexisting hearing disorders; age; polymorphisms in megalin, *ACYP2, TPMT, COMT*, and *XPC*; systemic inflammation	Air and bone conduction pure tone audiometry
Hematologic toxicities	1–24	95	—	Age, preexisting anemia/low hematocrit	Complete blood count, blood smear

a *Cumulative dose at which symptoms begin*.

b *Serum creatinine must be measured to calculate GFR or CCR but must not be considered alone because it does not directly reflect the level of renal function*.

*ACYP2, acylphosphatase 2; CCR, creatinine clearance rate; COMT, catechol-O-methyltransferase; ECOG PS, Eastern Cooperative Oncology Group performance status; GFR, glomerular filtration rate; TPMT, thiopurine S-methyltransferase; XPC, xeroderma pigmentosum, complementation group C*.

Cisplatin-induced neurotoxicity, experienced at grade ≥3 (by investigator choice of version of National Cancer Institute Common Terminology Criteria for Adverse Events) by ≤10% of patients (Table 1), affects mainly peripheral neurons—most likely via DNA damage–induced apoptosis of dorsal root ganglion neurons (45, 46)—and presents as sensory symptoms in the extremities (47, 48). Neurotoxicity resulting from peripheral nerve damage is irreversible in 30% to 50% of cases and progresses for ≤4 months after treatment (35, 49). Additionally, concurrent irradiation of the spinal cord, brain, brain stem, or cranial nerves can compound cisplatin-associated neurotoxicity and result in further functional deficit (50).

Ototoxicity is another highly distressing AE. Although its mechanism is not fully understood, ototoxicity arises predominantly from sensory hair cell death within the cochlea and is perpetuated by irradiation of and cisplatin-mediated damage to the cochlea (often neglected as an organ at risk) (38, 51, 52). Late ototoxicity is observed in up to two-thirds of cisplatin-treated patients, with ≤2% experiencing grade ≥3 events (equivalent to irreversible damage) (Table 1). Because some patients discontinue treatment due to hearing loss before it reaches grade ≥3 severity, the frequency and severity of ototoxicity vary and are likely underreported.

Cisplatin-induced nephrotoxicity is the result of a combination of factors. The kidney absorbs cisplatin at higher concentrations than other tissues (53, 54). In the kidney, cisplatin induces apoptosis via the activation of death receptors and tumor necrosis factor α-stimulated inflammatory response (39, 55). Nephrotoxicity cannot be completely prevented, even with adequate hydration regimens, and has been reported to occur as an acute AE in 7–75% of patients receiving the high-dose regimen (Table 1). Dehydration and electrolyte imbalance from chemotherapy can further contribute to cisplatin-associated late nephrotoxicity, leading to irreversible reduction in glomerular filtration rate (GFR) (40, 56, 57).

Acute gastrointestinal symptoms associated with cisplatin-based chemoradiotherapy include nausea, vomiting, mucositis/stomatitis, xerostomia, taste alteration, constipation, and diarrhea and have a remarkably high incidence (Table 1).

Cisplatin induces vomiting in >90% of patients within 24 h of treatment if not administered with adequate antiemetic prophylaxis (58–60). Even at a low dose (<50 mg/m^2^), cisplatin-induced vomiting occurs in 60–90% of patients (60). Nausea, vomiting, and mucositis further exacerbate dehydration, adding to the burden on patients' kidneys. Thus, physicians deem even low-grade (1/2) vomiting as unacceptable, especially in patients already experiencing dehydration.

Another compounding but poorly reported AE experienced by patients receiving platinum-based therapy is altered taste (typically metallic), which, combined with radiation-induced loss of taste, can lead to marked dysgeusia and consequently poor food intake and nutritional problems (61, 62). Additional radiotherapy-associated AEs such as dysphagia [which can cause feeding-tube dependence and ultimately aspiration pneumonia (63–65)], dry mouth, mucositis, and dermatitis can occur at high rates upon the addition of high-dose cisplatin to radiotherapy. A comprehensive list of toxicities associated with cisplatin and corresponding incidence rates is shown in Table 1.

## Absolute Contraindications vs. Borderline Unsuitability for Cisplatin

Since cisplatin's introduction as a chemotherapy agent in the late 1970s, a strong need has emerged to clearly define the patient population that is able to tolerate this treatment. For this purpose, studies investigating the correlations between baseline characteristics of patients and cisplatin-induced AEs have led to a consensus of absolute contraindications for cisplatin, which can be used to guide the treatment decision-making process.

Although it is often clear whether patients are absolutely or not at all contraindicated for high-dose cisplatin-based treatment, many patients fall in an in-between category of “borderline” unsuitability, for which the optimal treatment decision is not self-evident and must be made based on a physician's clinical judgment. Patients with conditions that may be worsened by the high-dose cisplatin regimen may experience QOL detriment while not reaching the recommended cumulative dose due to treatment interruption or cessation. The level of risk of sustaining irreversible cisplatin-associated toxicities should therefore be weighed against the potential benefit on a patient-by-patient basis. In many cases, these toxicities can be avoided if borderline-unsuitable patients are identified before treatment and preferentially redirected to alternative treatment regimens.

Absolute contraindications are outlined in Table 3 and include hypersensitivity to platinum, pregnancy/lactation, ECOG PS of ≥3, GFR of <50 mL/min or—only if GFR cannot be assessed—creatinine clearance rate of <50 mL/min (estimated by the Cockcroft-Gault formula), marrow disorders (in certain cases), preexisting hearing loss, neurological impairment, Child-Pugh score of B or C, New York Heart Association class III or IV congestive heart failure, and a severe hematologic condition (Table 3) (35, 41, 66–71). Notably, in regard to GFR of 50 to <60 mL/min, consultation with a nephrologist should be considered. Some clinicians offer dose-reduced cisplatin in these patients; this is a matter of discussion with the individual patient.

**Table 3:** Cisplatin unsuitability and borderline unsuitability criteria.

**Unsuitable**	**Borderline unsuitable**
Hypersensitivity to platinum	N/A
Pregnancy/lactation	N/A
Untreated chronic active hepatitis B or C	N/A
ECOG PS ≥ 3	ECOG PS ≥ 2
Preexisting renal dysfunction (CCR < 60 mL/min)	Any renal dysfunction and associated risk factors
Severe hearing impairment	Any hearing disorder and associated risk factors
Severe neurological disorders	Any neurological disorder (including peripheral sensory symptoms) and associated risk factors
Severe immunological condition (CD4-lymphocyte count <200/mm^3^, a detectable viral load, an acquired immunodeficiency syndrome)	Any immunological condition
Insufficient hydration	Poor hydration
Platelet count, < 100,000/mm^3^; neutrophil count, < 1,500/mm; or hemoglobin <9 g/dL; despite optimization attempt	Preexisting hematologic condition, anemia
NYHA class III or IV congestive heart failure	NYHA class I or II congestive heart failure in the presence of a left ventricular ejection fraction of ≤50%
N/A	Preexisting gastrointestinal condition, history of motion sickness and pregnancy-induced vomiting
N/A	Diabetes
N/A	Biological age ≥70 years
N/A	Bone marrow disorders
N/A	Having received platinum agents in induction chemotherapy

*CCR, creatinine clearance rate; ECOG PS, Eastern Cooperative Oncology Group performance status; N/A, not applicable; NYHA, New York Heart Association*.

In contrast, the borderline-unsuitable population includes patients with any current organ system dysfunction, especially any history of hearing, neurological, renal, hepatic, or cardiovascular disorders; a compromised immune system (e.g., HIV infection/AIDS); ≥20% weight loss; or a history of having received platinum agents in addition to taxanes in the induction setting for LA SCCHN (the only category 1–level, post-induction regimen for these patients and for selected primary tumor sites is radiotherapy alone; Table 3) (4, 35, 40, 68). In addition, some guidelines have recommended that patients older than 70 years and those with an ECOG PS of ≥2 or without objective evidence of euvolemia should not be treated with cisplatin (35, 40, 68). Reports have shown, however, that biological age is far more important and that patients who are treated only in accordance with chronical age may therefore be undertreated (72–74).

Although there is consensus on the risk factors for cisplatin toxicity in different indications, the clinical cutoff for unsuitability criteria for other cancer types should also be considered when treating patients with SCCHN. For example, guidelines for lung and urothelial cancer define ECOG PS of ≥2, New York Heart Association class II to III heart failure, and creatinine clearance rate (or GFR) of <60 mL/min as absolute contraindications for cisplatin (66–69). Finally, the combination of multiple comorbidities can have an additive effect on the risk of cisplatin toxicity (75).

## Risk Factors for Cisplatin-Induced Toxicities and Available Screening Methods

To assess whether a patient is contraindicated or borderline unsuitable for cisplatin, careful screening for comorbidities and evaluating the patient's current state of health and available psychosocial setting are necessary before prescribing cisplatin (35, 41).

Sensory symptoms in the extremities—such as weakness, tremor, numbness, paresthesia, and loss of position and vibration sense—are early predictors of severe cisplatin-induced neuropathy (47). Indeed, changes in vibration perception (assessable using a 128 Hz tuning fork on upper and lower limbs) and deep tendon reflex (assessable by standard deep tendon reflex test) have been shown to correlate with the severity of cisplatin-induced neuropathy (42). Furthermore, although known risk factors for chemotherapy-induced peripheral neurotoxicity such as history of drug and alcohol abuse, diabetes, high serum creatinine levels, and advancing age (Table 2) (76) have not been demonstrated to specifically and significantly increase the severity of cisplatin-induced toxicities (77), they should be considered when deciding on a treatment regimen for patients with LA SCCHN.

Key risk factors for cisplatin-induced ototoxicity are age and pre-existing hearing disorders (38). Because impaired hearing at baseline is not necessarily evident, it is not routinely or easily tested and thus should be evaluated by conduction audiometry before any cisplatin treatment (43).

Pre-existing renal disorders are the most important high-risk factors for cisplatin-induced nephrotoxicity and can be assessed by urinalysis and estimating GFR (10, 35, 38, 40). Measurement of creatinine clearance rate (see earlier recommendations) is not advised, because it has been shown to be dependent on body mass index and is often unreliable in practice due to faulty urine collection (78). Additional risk factors for cisplatin-induced nephrotoxicity are poor ECOG PS, regular use of non-steroidal anti-inflammatory drugs, advanced age, smoking, and diabetes (Table 2). Given that the risk of renal injury remains high despite all of these considerations, a model developed by Motwani et al. (79) to predict renal injury after the first cycle of cisplatin can be used.

Age is one of the most important risk factors for cisplatin-associated hematologic toxicities (80). A profound and prolonged decrease in neutrophil counts can be a serious issue and can lead to either a delay in cisplatin doses or failure to receive the total dose of 300 mg/m^2^. Low hematocrit (anemia) resulting from cisplatin treatment can increase the risk of severe hematologic AEs and should be measured in the blood (e.g., complete blood count or blood smear) before treatment. However, because anemia resulting from cisplatin therapy is rare and can be treated per ESMO guidelines (81), its risk should be weighed against the probability of cure.

Risk factors for chemotherapy-induced vomiting include age, sex (women are at higher risk), and history of motion sickness and/or pregnancy-induced vomiting (60). Patient age is a complex factor in the LA SCCHN treatment decision-making process and continues to be a topic of discussion. Specifically, although most young patients (≤55 years old) have good renal function and are thus optimal candidates for cisplatin treatment, their post-treatment QOL is of high consideration during the regimen selection process. To predict chemotherapy toxicity based on a patient's physiological age, a geriatric assessment can be performed using the scale developed by Extermann et al. (82).

## Preventive Measures to Avoid Cisplatin-Induced Toxicities

In addition to the level of difficulty associated with managing cisplatin-induced late toxicities, limited preventive management options exist, and careful patient selection remains the most effective method to reduce overall risk. Because of the high incidence of toxicities, all available prophylactic measures should be applied when treating eligible patients with cisplatin, and patients should be closely monitored during and after therapy. Furthermore, patients with SCCHN should be managed by a multidisciplinary team (4, 5, 41).

Some studies suggest that accelerating the infusion time of cisplatin can exacerbate the severity of toxicities. A study comparing a 2 vs. 24 h infusion time, performed before the development of efficient antiemetic treatment, revealed significantly less emetic toxicity with the 24 h infusion of 50 or 100 mg/m^2^ and pretreatment hydration (83); hence, infusion times shorter than the recommended 6–8 h are not advisable. Furthermore, studies have suggested that lowering the dose of radiation to the pharyngeal constrictors and larynx to <50 Gy may reduce the risk of swallowing complications such as long-term dysphagia (84, 85); however, such a reduction is inadvisable because it may also lower the probability of tumor control.

Additionally, any concomitant neurotoxic, ototoxic, and nephrotoxic drugs should be strictly avoided (including aminoglycosides, furosemide, and non-steroidal anti-inflammatory drugs) (40, 86). Numerous studies have suggested that thiol compounds (e.g., glutathione), vitamin E, and anticonvulsants (e.g., gabapentin, pregabalin) can have a neuroprotective effect (87). Thiosulfates have also been used against ototoxicity (38). However, these strategies have failed to provide conclusive evidence of the prevention of neurotoxicity and ototoxicity secondary to cisplatin treatment and are not universally recommended (87). Although no effective measures are available to fully prevent cisplatin-associated ototoxicity, its occurrence can be reduced by administering reduced-dose radiotherapy directly to the cochlea; the threshold for ototoxicity was found to be 10 Gy (88).

Dehydration through nausea and vomiting is one of the most serious and unpleasant side effects of chemoradiotherapy with cisplatin, and keeping a patient sufficiently hydrated is an effective and widely used method to counteract associated nephrotoxicity (40). The recommended hydration regimen consists of a continuous infusion of a normal saline solution starting 12 h before cisplatin administration and ending ≥1 day after treatment (40). To avoid further nephrotoxicity and dehydration, triple or quadruple antiemetic therapy consisting of a 5-hydroxytryptamine-3 (serotonin) receptor antagonist, a neurokinin 1 receptor antagonist, and dexamethasone (with or without olanzapine)—per Multinational Association of Supportive Care in Cancer/ESMO and American Society of Clinical Oncology guidelines—and sufficient doses of magnesium (40–80 mmol/cycle), should always be administered concomitantly with cisplatin (40, 58, 59). Conversely, studies of the effectiveness of mannitol diuresis in preventing nephrotoxicity have shown contradictory results; hence, caution should be exercised when considering it as a protective measure (89–91).

## Baseline Characteristics Dictating a Qol-Centric Approach

Recently, the long-term impact of treatment-related toxicities on the QOL of responders has come into focus in populations with a favorable prognosis. Specifically, overall, patients with HPV-positive OPC have a very good prognosis and tend to be younger and have little or no smoking history, fewer comorbidities, and improved locoregional control and OS compared with patients with HPV-negative tumors (92–94). Results from two phase III trials in patients with HPV-positive OPC suitable for cisplatin (RTOG 1016 and De-ESCALaTE) reinforced the high-dose cisplatin plus radiotherapy regimen as the standard of care for this patient population in terms of OS (95, 96). For the overall population, cetuximab plus radiotherapy remains a guideline-recommended treatment option, and has demonstrated a higher response rate, longer disease-free progression, and longer OS vs. radiotherapy alone (4, 5). The results of the TROG 12.01 study, which is comparing the combination of either cetuximab or weekly cisplatin with radiotherapy, are awaited (97). Finally, lowering the radiotherapy dose is being explored as a method of preventing detriment to QOL in patients with HPV-positive disease. It should be noted that the recently presented NRG-HN002 trial demonstrated that the addition of cisplatin to 60-Gy intensity-modulated radiation therapy in HPV-positive patients provided additional tumor control (98).

## Alternative Treatment Options for Cisplatin-Unsuitable and Borderline-Unsuitable Patients

Patients with LA SCCHN who are strictly contraindicated to receive cisplatin depend on alternative treatments and/or future advances in this field, and those who are either cisplatin borderline unsuitable or have favorable prognoses may strongly benefit from them. To find the optimal regimen for each patient, the benefits and disadvantages of alternative regimens need to be carefully balanced. Despite the lack of phase III trials demonstrating the efficacy of alternative treatments vs. high-dose cisplatin–based chemoradiotherapy in the overall LA SCCHN population, several non-cisplatin regimens are currently recommended by international guidelines, of which only two do not include a platinum agent: radiotherapy plus either 5-fluorouracil [5-FU] and hydroxyurea or cetuximab (4, 5).

Data from a recent meta-analysis by the Meta-Analysis of Radiotherapy in Squamous Cell Carcinomas of Head and Neck (MARCH) Group demonstrated that concomitant chemoradiation is significantly superior to altered fractionation radiotherapy alone in terms of OS (99). Although no direct comparison between hyperfractionation (which seemed superior to other altered fractionation methods) and concomitant chemoradiation has been made, these results indicate that patients with LA SCCHN should not be treated with conventionally fractionated radiation alone (99).

### Low-Dose Weekly Cisplatin Plus Radiotherapy

Because of the high incidence of AEs associated with high-dose cisplatin, clinical practice has focused on splitting the dose of 100 mg/m^2^ every 3 weeks into weekly doses of 20–50 mg/m^2^, which also allows the physician to monitor the patient weekly and hence potentiates supportive care. Weekly cisplatin 40 mg/m^2^, the most commonly used weekly regimen, has not been evaluated in a large randomized study vs. concurrent high-dose cisplatin chemoradiotherapy or radiotherapy alone and is currently supported by a category 2B level of evidence in international guidelines (4). A retrospective analysis suggested a potentially reduced efficacy with this regimen (median OS, 1.9 vs. 4.3 years for weekly 40 mg/m^2^ vs. high-dose cisplatin, respectively) (11). An evaluation of single-dose administration of 100 mg/m^2^ of cisplatin compared with a split dose of 50 mg/m^2^ per day on two consecutive days found no significant difference in OS between these two regimens in patients with SCCHN (100). Reported compliance rates with cisplatin 40 mg/m^2^ weekly are contradictory: one study (14) showed similar compliance with both regimens, whereas three studies (including COMPLY in the real-world setting) showed better compliance with high-dose than with weekly cisplatin (8, 15, 16).

Weekly cisplatin doses of <40 mg/m^2^ have failed to show superiority or non-inferiority to radiotherapy alone or to high-dose cisplatin in terms of efficacy and treatment compliance in multiple trials (8, 101, 102). For example, a phase III study conducted by the Head and Neck Intergroup (102) reported a median OS of 11.8 months with weekly cisplatin 20 mg/m^2^ plus radiotherapy and of 13.3 months with radiotherapy alone. In the real-world, retrospective, observational study COMPLY, compliance was lower with cisplatin 30 mg/m^2^ weekly than with high-dose cisplatin; toxicity was comparable (8).

Overall, weekly cisplatin treatment is precluded by the same contraindication panel as the high-dose schedule. Furthermore, because the level of toxicity risk with weekly cisplatin regimens has not been investigated in patients who are considered borderline unsuitable for high-dose cisplatin, this treatment cannot be said to circumvent any of the detriments of the high-dose schedule in this patient population.

### Alternative Chemotherapy Agents in Combination With Radiotherapy

Efforts made to find less-toxic chemotherapeutic agents resulted in the development of carboplatin—a second-generation platinum-based drug with a mechanism of action similar to that of cisplatin but with a different chemical structure and a somewhat milder toxicity profile. Indeed, a meta-analysis (103) of 12 studies—published between 1995 and 2013—in patients (*n* = 1,165) with LA SCCHN showed improved 5-year OS with cisplatin. Toxicity profiles of the two agents were similar, although fewer gastrointestinal and renal toxicities but more frequent hematologic toxicities were observed with carboplatin. Because of the lack of randomized phase III trials, however, treatment of LA SCCHN with carboplatin and concurrent radiotherapy is not currently considered an evidence-based option in LA SCCHN (104). Carboplatin in combination with 5-FU and radiotherapy (predominantly used in France) has demonstrated improved OS and locoregional control compared with radiotherapy alone in two phase III trials (105, 106) and is recommended with a category 1 level of evidence (4). The most frequent grade 3 treatment-related AE with carboplatin and 5-FU is mucositis; other grade ≥3 toxicities include fever, renal toxicity, skin reaction, and altered liver enzyme function (107). As with cisplatin, patients need to be fit to receive carboplatin plus 5-FU; this regimen is thus an alternative option for patients with contraindications to cisplatin specifically (e.g., reduced renal or hearing function) who have a good PS. Therefore, non-platinum-based anticancer agents in combination with radiotherapy are needed for the treatment of LA SCCHN. A phase III trial comparing 5-FU + mitomycin C + hyperfractionated accelerated radiation therapy (HART) with cisplatin + mitomycin C + HART demonstrated no significant differences in OS, progression-free survival (PFS), or locoregional control. Chemoradiation with weekly cisplatin + 5-FU or mitomycin C + 5-FU showed excellent adherence rates and can easily compete with other concurrent chemoradiation schedules, including induction with docetaxel + cisplatin + 5-FU followed by radiation (108).

### Targeted Therapies in Combination With Radiotherapy

A major recent advance in the LA SCCHN field was the development of cetuximab, an immunoglobulin G1 monoclonal antibody targeting the epidermal growth factor receptor. The effects of adding cetuximab to radiotherapy were studied in a randomized phase III trial by Bonner et al. (109), who reported a significant increase in median PFS (17.1 vs. 12.4 months), median OS (49.0 vs. 29.3 months), and median duration of locoregional control (24.4 vs. 14.9 months) in the cetuximab plus radiotherapy arm vs. radiotherapy alone. The 5-year survival rate was also considerably higher with cetuximab plus radiotherapy (45.6 vs. 36.4%) (110). On the basis of these results, cetuximab became the first targeted therapy approved by the US Food and Drug Administration for LA SCCHN in 2006 (111) and is now recommended by international guidelines (4, 5).

In contrast with cisplatin, no exacerbation of in-field mucositis and dysphagia and no evidence of ototoxicity, neurotoxicity, or nephrotoxicity were observed with the addition of cetuximab to radiotherapy at the end of the trial (Table 4) (109); hence, no reduction in the cetuximab dose is needed in patients with preexisting reduced renal function (111). Also unlike cisplatin, cetuximab does not significantly aggravate radiation dermatitis (109). However, when cetuximab is combined with radiotherapy, different aspects of the skin rash (e.g., crusting) can appear, which have been referred to as bioradiation dermatitis (114, 115). Furthermore, cetuximab is also associated with acneiform rash, a distinct skin rash characteristic of epidermal growth factor receptor inhibition that is severe in only ≤17% of patients. Acneiform rash can be prophylactically treated, resolves within weeks, and correlates with improved OS (109, 110). Randomized phase III data indicate that QOL is not affected by the addition of cetuximab to radiotherapy, despite the associated dermatologic AEs (116). Furthermore, the addition of cetuximab to radiotherapy does not appear to significantly increase the incidence of common radiotherapy-associated toxicities, including mucositis, xerostomia, dysphagia, pain, weight loss, and performance status deterioration (116). Notably, toxicity profiles of cisplatin in combination with radiotherapy and cetuximab in combination with radiotherapy have been shown to differ. Hematologic, renal, and gastrointestinal toxicities appear to occur more frequently with cisplatin combined with radiotherapy, and cutaneous toxicity and the need for nutritional support occur more frequently with cetuximab combined with radiotherapy (17). Differences in the toxicity profile of cisplatin vs. cetuximab in combination with radiotherapy were also confirmed in the RTOG 1016 study in patients with HPV-positive OPC (95), which showed a similar overall rate of grade ≥3 AEs but increased rates of nephrotoxicity, ototoxicity, and bone marrow suppression with cisplatin plus radiotherapy (95). As expected, rash was more frequent with cetuximab treatment (95). Similarly, in the De-ESCALaTE study in patients with low-risk HPV-positive OPC, the cisplatin arm showed comparable rates of all- and high-grade AEs and long-term dysphagia and no difference in QOL, but significantly more serious AEs per patient were observed (96). The tolerability of these regimens may be different in HPV-negative patients, who are older and tend to have more comorbidities (92–94).

**Table 4:** Frequency of cetuximab bioradiotherapy–associated acute toxicities in LA SCCHN (17, 109, 112, 113).

**Cetuximab/radiotherapy-induced toxicity, %**	**Any grade**	**Grade ≥3**
Mucositis/stomatitis	58–93	0–73
Dermatitis	42–86	0–44
Dysphagia	65	14–26
Radiation skin injury	NR	23
Infection	13	1–23
Appetite	NR	18
Acneiform rash	64–87	17
Gastrointestinal	26	3–14
Hematologic	3	1–14
Weight loss	84	11
Dry skin	68	9
Diarrhea	19	2–9
Pain	28	6
Dehydration	25	6
Xerostomia	72–77	5
Constipation	35–68	5

*LA SCCHN, locally advanced squamous cell carcinoma of the head and neck; NR, not reported*.

Cetuximab in combination with radiotherapy is an efficacious treatment option in LA SCCHN and can be used as an alternative in patients who are unsuitable to receive high-dose cisplatin plus radiotherapy. The randomized phase III trial by Bonner et al. that is discussed above included a large number of patients who would be considered fit and cisplatin eligible. There are no phase III data showing benefit of cetuximab in combination with radiotherapy in the non–platinum-eligible population. Notably, combinations of cetuximab with non-cisplatin chemoradiotherapy agents, such as carboplatin plus paclitaxel or 5-FU, are currently being investigated and have thus far shown promising results (117–119). A recent study demonstrated that the addition of concurrent carboplatin and fluorouracil to cetuximab (three cycles) + radiotherapy resulted in improved PFS and locoregional control. However, the gain in OS was not significant (119).

## Emerging Immunotherapy Agents Plus Radiotherapy

Immune checkpoint inhibitors are hypothesized to synergize well with radiotherapy (120). Immune checkpoint inhibitors (e.g., avelumab, nivolumab, pembrolizumab) are under investigation in combination with radiotherapy, chemoradiotherapy, or cetuximab/radiotherapy for LA SCCHN (36, 120–124), and any resulting chemotherapy-sparing regimens could be useful in cisplatin-ineligible patients. For example, avelumab plus cetuximab plus radiotherapy is being evaluated in patients with LA SCCHN in the ongoing phase III REACH study (125). Because no mature results are available for immune checkpoint inhibitors in LA SCCHN, these agents should not yet be used outside of the clinical trial setting.

## Conclusions

High-dose cisplatin-based concurrent chemoradiotherapy is the preferred treatment for fit patients with LA SCCHN. Because high-dose cisplatin is associated with a considerable number of toxicities, it is not recommended for patients aged >70 years or with an ECOG PS of ≥2 (35). Preexisting comorbidities, such as neurological disorders, renal impairment, and hearing loss, can be exacerbated irreversibly by cisplatin treatment and are tentative or absolute contraindications for cisplatin treatment. In addition to absolute cisplatin-unsuitable patients, patients with a high risk of treatment non-adherence for concurrent high-dose cisplatin chemoradiotherapy are defined herein as borderline unsuitable.

Alternative chemotherapy regimens and administration schedules, targeted agents, and emerging immunotherapies provide possible treatment options for cisplatin-contraindicated or borderline-unsuitable patients with LA SCCHN. HART alone could also be a reasonable alternative for cisplatin-contraindicated patients (126). However, few of the previously-mentioned options have been tested for non-inferiority vs. high-dose cisplatin in randomized, phase III trials in the overall LA SCCHN population. Cetuximab is an approved non-platinum agent that is recommended for combination with radiotherapy in the treatment of LA SCCHN. This regimen has demonstrated good locoregional control and survival outcomes in patients with this tumor type and is thus a suitable treatment alternative for patients who are unlikely to tolerate high-dose cisplatin.

The reference tools provided in this review should facilitate the treatment decision-making process in LA SCCHN for oncologists and healthcare professionals before prescribing high-dose cisplatin chemoradiotherapy for their patients.

## Author Contributions

SP, FS, MA, SS, AC, VL-V, and LL contributed equally to manuscript development in terms of manuscript preparation, manuscript review, and final approval.

### Conflict of Interest

SS discloses employment with Merck KGaA, Darmstadt Germany. The remaining authors declare that the research was conducted in the absence of any commercial or financial relationships that could be construed as a potential conflict of interest.
